# Mindful Parenting Group Intervention for Parents of Children with Anxiety Disorders

**DOI:** 10.1007/s10578-023-01492-2

**Published:** 2023-01-23

**Authors:** Robyn Farley, Natalja A. Nabinger de Diaz, Lisa Marie Emerson, Gabrielle Simcock, Caroline Donovan, Lara J. Farrell

**Affiliations:** 1 Griffith University, Gold Coast, Australia; 2 Griffith University, Mount Gravatt, Australia; 3 University of Canterbury, Aotearoa, New Zealand; 4 School of Applied Psychology, Health Building (G40), Parklands Drive, Southport, QLD 4222 Australia

**Keywords:** Mindful parenting interventions, Anxiety disorders, Children, Parenting, Mindfulness, Intervention

## Abstract

Parenting behaviour and rearing style contribute to the intergenerational relationship between parental and child anxiety. Current psychological interventions for child anxiety typically do not adequately address parental mental health, parenting behaviours or the parent–child relationship. The current pilot study examines the effectiveness of a mindful parenting intervention (MPI) for parents of young children with clinical anxiety. It was hypothesised that the intervention would be associated with improvements in parental stress, mental health, and mindfulness, and a reduction in child clinical anxiety symptoms. Twenty-one parents of children aged 3–7 years diagnosed with anxiety disorders participated in an 8-week group MPI program that aimed to increase their intentional moment to moment awareness of the parent–child relationship. Parental (anxiety, depression, hostility, stress, burden, mindfulness, mindful parenting) and child (anxiety diagnoses, anxiety severity, comorbidities) outcomes were assessed at pre- and post-intervention, and at 3-month follow-up. Parents reported a significant increase in mindful parenting and a significant reduction in parent–child dysfunctional interaction, but no change in mental health symptoms. There was a significant reduction in parent-rated child anxiety symptoms, severity of child anxiety diagnosis and number of comorbid diagnoses at post and 3-month follow-up. Limitations include a lack of waitlist control, small sample size, and participants were largely mothers, from intact families and highly educated. There was attrition of 43% and outcomes were predominantly self-report. MPIs offer a novel and potentially effective method of increasing mindful parenting, decreasing dysfunctional parent–child interactions, reducing parenting stress and might also be an effective early intervention for indirectly decreasing young children’s clinical anxiety symptoms. Larger-scale controlled trials of MPIs are needed.

## Introduction

Child anxiety disorders are common, can be impairing, and persistent [1]. They have a median onset of 6 years [2], and show stability over early childhood [3]. One study found that 34% of children with an anxiety disorder at age 3 years continued to meet criteria for the diagnosis at age 6 years [4]. Furthermore, child anxiety disorders predict later mental health conditions and impairment in functioning [5]. However, only 5% of young children with clinical anxiety receive intervention or support, despite the potential for early intervention to lessen the impact on children’s development [6].

### Parenting and Child Anxiety

Various parental rearing and family factors are important risk and protective factors of child anxiety disorders [7–11]. Anxiety disorders tend to run in families, with children up to seven times more likely to experience an anxiety disorder if their parents also have an anxiety disorder than if parents do not; and this risk is elevated further when both parents suffer from anxiety [12–17]. Additionally, parents of anxious children experience heightened stress and distress compared to parents of non-anxious children [18].

Parent anxiety, depression and stress impact negatively on sensitive maternal caregiving, including decreased parental warmth, sensitivity, and responsiveness, as well as higher intrusiveness, punitiveness, and harsh, over reactive and ineffective discipline strategies [19–24]. Highly stressed parents often engage in parenting practices that can negatively impact children (e.g., authoritarian parenting, overprotection and higher child neglect and abuse potential (Miragoli, 2018, Chow, 2015, Carapito, 2020). Moreover, parental psychopathology [3, 25], stress and strain [6], and negative parental rearing behaviours [3, 26–28] are associated with pre-schooler anxiety, predictors of subsequent anxiety, as well as predictors of poor treatment response amongst school-aged children. Conversely, longitudinal studies reveal that sensitive, maternal caregiving predict lower rates of anxiety disorders in 4 to 6-year-old children, suggesting that these factors play an important protective role at this developmental stage [29, 30].

There is empirical support for developmentally tailored individual child-focused cognitive behavioural therapies (CBT) for anxiety. However, for 30 to 50% of children this treatment fails to alleviate anxiety symptoms [31, 32], and there is a relapse rate of around 10%, particularly among younger children with co-occurring externalising disorders (Levy et.al., 2022). Thus, there remains room for improvement for young children with anxiety disorders and their families by addressing systemic family factors may improve longer-term remission rates.

Metanalytic reviews did not find that parental involvement in treatment improved efficacy for CBT amongst school aged children [33]. Although it is not clear why parental involvement does not improve treatment for anxious children, however, there is some evidence that this line of research should continue to be pursued. For example, one study examining family-based treatment for childhood anxiety found parent involvement yielded higher remission rates for younger children (7–10 years) than older children [34]; and therefore we should investigate novel parental involvement interventions for young children. Even in studies where parents participate in CBT for their anxious child, interventions typically have not addressed parents’ own mental health and stress. There is a need to further explore the role of parental stress and psychopathology in novel treatments due to anxiety running in families, the high stress of parenting anxious children, the bidirectional relationship between parent and child psychopathology, and the adverse effects of parent psychopathology and stress on parenting behaviours. It is possible that reducing parental stress and psychopathology may decrease the impact of child anxiety on the children themselves and their families.

### Mindful Parenting and Mindful Parenting Interventions (MPIs)

Mindful parenting offers a novel approach to supporting parents of anxious children, with therapeutic benefits for the dyad. Dispositional mindfulness is intrapersonal and refers to a person’s capacity to pay attention to the present moment with an open and nonjudgmental attitude [36, 37]. Those with higher levels of dispositional mindfulness report experiencing more positive emotions, adaptive emotional regulation, greater social connectedness, higher satisfaction in interpersonal relationships, stable cortisol levels and reduced depression, anxiety and stress compared to those with low levels of dispositional mindfulness [36, 38–42]. Mindful parenting is interpersonal and refers to the intentional moment to moment awareness of the parent–child relationship; by listening with full attention to the child, cultivating emotional awareness and self-regulation in parenting, and bringing compassion and nonjudgmental acceptance to the self and child to enhance parent–child interactions [43]. As a disposition, mindful parenting can be cultivated through training with a family of MPIs recently developed, aiming to reduce parenting stress and over-reactivity, and to enhance dispositional mindfulness (intrapersonal) and mindful parenting (interpersonal) [44].

Numerous studies have investigated the benefits of MPI’s for parents of children on the autism spectrum [45], with Attention Hyperactivity Deficit Disorder (ADHD) [46, 47], developmental delay [48], and depression [49], as well as with parents of non-clinical/community samples of children [50]. For parents, MPIs have produced clinically significant reductions in psychopathology, parental stress, ADHD symptoms, maladaptive cognitive emotion regulation, and unhelpful beliefs regarding child anxiety [51]. Parental MPI’s also reduced children’s psychopathology [47, 49], ADHD symptoms and behavioural problems [52, 53].

Research to date has not examined the effectiveness of MPIs for parents of children with anxiety disorders. Given the known high rates of stress and psychopathology experienced by parents of anxious children [18] and the associations between parent mental health, parenting behaviour and child anxiety [27], a MPI may be an efficacious early intervention for reducing parental stress and mental health and (indirectly) improving outcomes for young children with anxiety.

### The Current Study

The aim of this study was a preliminary investigation into the effectiveness of an 8-week MPI for parents of clinically anxious children aged 3 to 7 years on outcomes of parental mental health, parenting stress, burden, mindfulness and mindful parenting at post-intervention, and at 3-month follow-up. We hypothesised that parents would report significant improvements in these outcomes following the MPI, with gains maintained at 3-month follow-up.

A secondary aim was to examine whether young children whose parents attend the MPI would benefit (indirectly). We hypothesised that children would show significant improvements in anxiety symptoms, diagnostic severity and comorbidity, with gains maintained at 3-month follow-up.

## Methods

### Participants

Participants were 21 parents (95.2% mothers, *M*_age_ = 39.71, *SD* = 5.64; age range 31–53 years) and their clinically anxious children (47.6% female, *M*_age_ = 5.2 years, *SD* = 1.4; age range 3–7 years) who volunteered to participate in a pilot study examining the effects of an 8-week MPI. Children and their parents were recruited via advertising in kindergartens, childcare centres, primary schools, and Griffith University, in southeast Queensland, Gold Coast, Australia between June 2019 and February 2020.

Inclusion criteria were: (1) the child presented with a DSM 5 (APA, 2013) primary anxiety disorder diagnosis; (2) caregiver (or person in the role of a parent) is willing to cease concurrent parenting courses or psychotherapy (child or caregiver) for the duration of study; (3) stable dose of medication for parent and/or child for the duration of study; (4) at least one parent is willing to attend sessions; and (5) parental proficiency in English. Exclusion criteria were: (1) the presence of a non-anxiety primary diagnosis (e.g., Autism, ADHD, or depression; (2) parent having current or recent (within the last year) substance dependence; (3) parent at current moderate to high risk of self-harm or suicide; and (4) a current substantial risk of child abuse in the family. Table 1 provides demographic information regarding all participants.

**Table 1:** Demographics of parents and children (n = 21)

Demographic variable	Entire sample *n* (%)
Parent gender^a^	
Mothers	20 (95.2)
Fathers	1 (4.8)
Ethnicity/nationality	
Australian	19 (90.5)
Other^b^	2 (9.5)
Parent marital status	
Married/De facto	16 (76.2)
Separated/divorced	3 (14.3)
Widowed	2 (9.5)
Parent education	
Year 10	1 (4.8)
Highschool	2 (9.5)
Diploma/trade	10 (47.6)
Undergraduate degree	5 (23.8)
Post graduate degree	3 (14.3)
Household income	
< 50 K	4 (19.0)
51–100	5 (23.8)
> 101	12 (57.1)
Child gender	
Female	10 (47.6)
Male	11 (52.4)
Child living arrangements	
Both parents	13 (61.9)
With mother	4 (14.3)
Grandparents	1 (4.8)
Shared care	3 (14.3)
Parent age—*M *(*SD*)	39.71 (5.64) 31–53
Child age—*M *(*SD*)	5.2 (1.4) 3–7

^a^Data is presented in count (%), except for age (presented in mean and standard deviation

^b^One family South African and one family from USA, K = 1000: $ = Australian Dollar

**Fig. 1:**
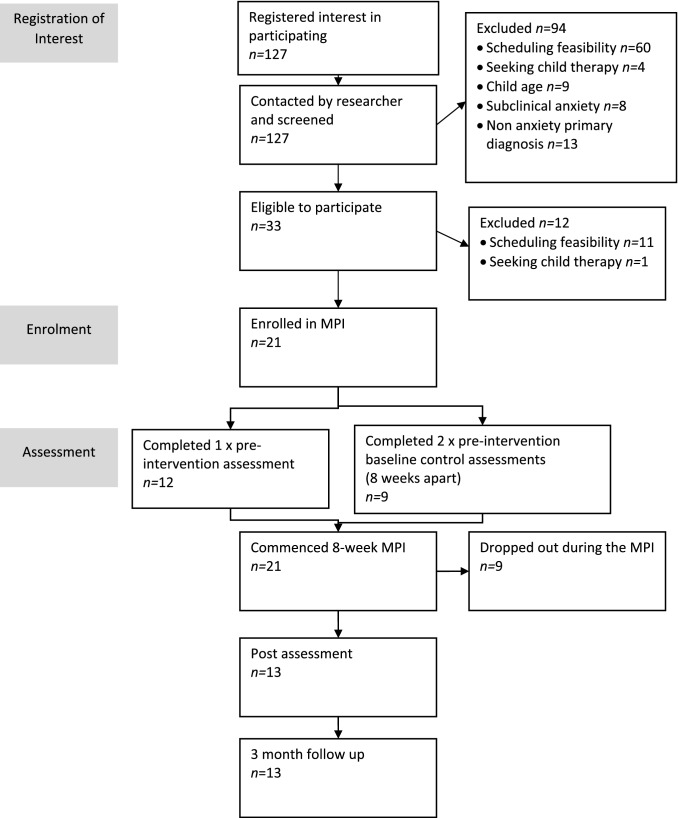
Flow of participants from registration of interest to follow-up assessment

Of the 21 parents enrolled, a subsample of 9 parents completed two baseline measures 8-weeks apart prior to the intervention (to assess the stability of these measures prior to the start of the MPI), post-intervention, and at 3-month follow-up, as can be seen in the CONSORT diagram (Fig. 1). Attrition was due to high levels of anxiety about being part of a group (n = 2), unable to find a carer for child(ren) while they attended the sessions (n = 6), and familiarity with the course content (n = 1). Due to the declaration of the COVID-19 pandemic during this study, the baseline phase was abandoned to ensure all participants would receive the face-to-face MPI, and thus the remainder of the sample (*n* = 12) completed only one pre-intervention assessment (rather than two) prior to receiving the MPI, as well as at post-intervention and at three-month follow-up.

### Measures

#### Parent Measures

##### Parental Mental Health

Four Brief Symptom Inventory (BSI) [54] subscales were used to assess parents mental health and hostility, including the depression (6 items, scores ranging from 0 to 24), anxiety (6 items- scores ranging from 0 to 24), phobia (5 items-scores ranging from 0 to 20), and hostility (5 items-scores ranging from 0 to 20) subscales The BSI subscales are rated on a on a 4-point Likert scale (0 = *not at all* to 4 = *extremely*). Total anxiety (sum of the phobia and anxiety subscales) ranged from 0 to 44 with higher scores signifying higher anxiety. Scores above the 85^th^ percentile indicates elevated levels of depression, hostility, and anxiety. The BSI has good test–retest reliability (0.68 to 0.91) and excellent internal consistency (0.95, [54, 55]. Cronbach’s α was 0.83 for both the total anxiety and depression subscales and 0.85 for the hostility subscale.

##### Parenting Stress

Parenting stress was measured with the Parenting Stress Index-Short Form (PSI-SF) [56], a 36-item inventory scored on a 5-point Likert scale (0 = *strongly disagree* to 5 = *strongly agree*). The PSI-SF contains three subscales (12 items each and scores ranging from 0 to 60): parental distress (distress from parental mental health, marital conflict or life restrictions/burden of parenting), dysfunctional parent–child interaction (parental dissatisfaction with parent–child relationship and sense that their child is acceptable/unacceptable) and difficult child (degree to which child is difficult/easy to parent, manages/does not manage to regulate emotions). Scores ≥ 85th percentile indicates elevated levels of parenting stress. The short form shares the validity of the full length version (correlation coefficient = 0.94) and test retest reliability of the full length (with *r* = 0.95) [56]. Cronbach’s α for this sample was 0.92 for total parent stress, 0.90 for dysfunctional parent–child interaction, 0.91 for difficult child and 0.87 for parental distress.

##### Parental Burden

The Burden Assessment Scale (BAS) [57] was used to measure the degree to which the child’s mental health disrupted family life and routines. Parents rated 21 items on a 5-point Likert scale (1 = *not at all* to 5 = *very much*). Scores were summed to produce a total burden score that ranged 0–105, with higher scores signifying greater burden. The BAS has good reliability with a Cronbach’s α of 0.93 [58]. The Cronbach’s α for the BAS in the current study was 0.92.

##### Mindful Parenting

The total score of the Interpersonal Mindfulness in Parenting scale (IM-P) [59] was used to assess mindful parenting. Parents rated each of the 31 items on a 5-point Likert scale (1 = *never true* to 5 = *always true*). Scores are summed to produce a total score that ranges from 0 to 155, with higher scores indicating higher mindful parenting. The IM-P has good internal reliability and construct validity in previous studies [59]. The Cronbach’s α for the IM-P in the current study was 0.91.

##### Dispositional Mindfulness

The Five Facet Mindfulness Questionnaire (FFMQ) [60] was used to assess dispositional mindfulness. Parents rated each of 39 items on a 5-point Likert scale (1 = *never true* to 5 = *always true*). Items are summed to produce a total score that ranges from 0 to 195, with higher scores reflecting greater dispositional mindfulness. FFMQ has good construct validity and internal reliability amongst meditating and non-meditating samples with Cronbach’s α ranging from 0.81 to 0.92 [61].The Cronbach’s α for the FFMQ in the current study was 0.92.

#### Child Measures

##### Child Diagnoses

At baseline the Anxiety Disorders Interview Schedule for Children- Parent Version (ADIS-P) [62] was used for anxiety and non-anxiety diagnoses. The ADIS-P is a semi-structured clinician-rated diagnostic interview, administered to the parent via telephone interview, a valid method of assessment (Lyneham, 2005), and the reliability of diagnoses has been established in preschool aged children [63]. Diagnoses are assigned a clinician severity rating (CSR: 0 = *not present* to 8 = *very severe*), with scores of four and above indicating a clinical level of symptoms. The ADIS-P has well established psychometric properties and high levels of interrater and test–retest reliability [64]. The Cronbach’s α amongst pre-schoolers has been found to range from 0.77 to 0.86 [63]. ADIS-P pre-intervention interviews and CSR ratings were conducted by RF and NN, and all post-intervention interviews and CSRs were conducted by independent masters level clinicians. All clinicians were trained in the ADIS-P administration and diagnoses were determined in supervision meetings with the primary supervisor (LJF).

##### Child Anxiety Symptoms

Due to participants’ age range, parents completed either the Preschool Anxiety Scale-Revised (PAS-R) [65] which is a 34-item scale (for children aged 3–5 years), or the Spence Children’s Anxiety Scale-Parent Version (SCAS) [66], which is a 45-item scale (for children aged 6–7 years). Both assess anxiety symptoms, and t-scores were used to ensure equivalence. Parents rated items on a 5-point Likert scale (0 = *not at all true* to 4 = *very often true*). The PAS has well established reliability (ranging from 0.85 to 0.88) [31] with Cronbach’s α 0.88 for the overall scale. The SCAS shows good internal consistency in clinical and non-clinical samples and convergent validity with related measures [67]. Cronbach’s α for this sample was 0.86.

### Procedure

Ethical approval was gained through the Griffith University Human Research Ethics Committee (GU Ref No: 2018/894). Families from the Gold Coast, Southeast Queensland, Australia self-referred to the trial in response to advertisements and were screened via telephone interview by RF (registered clinical psychologist) to determine eligibility. If inclusion criteria were met, pre-intervention telephone diagnostic interviews (ADIS-P) were conducted. Ineligible participants were referred to other appropriate services. All parents gave verbal consent prior to telephone interviews/screens and provided online informed consent before study commencement.

### Mindful Parenting Intervention

Parents participated in an 8-session group MPI in-person at the Griffith University Psychology Clinic (Gold Coast). The intervention was adapted from the manualised MPI program by Bögels & Restifo (2015) with minor modifications including; 8 weekly 2.5-h group sessions (rather than 8 weekly 3-h sessions), parents were recruited from the community (rather than treatment clinics), formal in-session meditation practices (including body scans, sitting meditations and yoga and walking practices) were shortened (from 30–45 min to 15–30 min); and a half day of mindfulness occurred between the 4^th^ and 5^th^ session (rather than a self-guided home practice ‘day of mindfulness’). Session topics included: (1) Automatic pilot, (2) Beginner’s mind, (3) Reconnecting with the body (4) Responding versus reacting, (5) Parenting patterns and schemas, (6) Conflict and parenting, (7) Love and limits and (8) Are we there yet? A mindful path through parenting. Additionally, parents attended a 1.5-h booster session, 4 weeks following program completion. All sessions consisted of an overview of the session theme, discussion of home practice, formal meditation practices followed by group inquiry and mindfulness/visualisation exercises.

Three separate groups were conducted with 4 parents in the first and second group and 13 parents in the third group. Parents were provided with weekly handouts summarising session themes, and an app with audio recordings of home practice. Parents were encouraged to complete approximately 45 min of home practice daily, including formal meditations and the application of mindful parenting in their interactions with their child. Overall, the duration of the intervention was 25.5 h (excluding home practice).

### Integrity of Intervention

The following quality assurance processes-maintained fidelity of the intervention: The program was led by author RF who has a personal mindfulness practice and had completed teacher training in mindfulness-based interventions. The MPI was co-facilitated by author NN. Both authors have experience working with parents and completed a workshop with the manual author (Susan Bögels). The facilitators adhered to the manualised protocol and attended bi-weekly supervision sessions during the delivery of the first round of the program with a Senior Clinical Psychologist and trained MPI facilitator (LME). The group sessions were also audio-recorded and 10% were randomly reviewed for integrity (LME) for the first round and discussed in supervision.

### Overview of Data Analyses and Data Screening

All statistical analyses were conducted using SPSS version 27. This study used a pre assessment 8-week monitoring phase for a proportion of the sample (*n* = 9) to control for within subjects’ effects of time, and to establish stability of parent mental health, parental stress, parent burden, child anxiety severity and mindful parenting. Paired samples t-tests were conducted for outcome measure for this sub-group from pre-baseline to post-baseline (8 weeks later).

To assess overall intervention outcomes for the entire sample, a series of one-way repeated measures ANOVAS were conducted to compare the effect of the MPI across three time points (pre-intervention, post-intervention and 3-month follow-up). ANOVA results were computed for completers and an intention to treat (ITT) sample. For the ITT sample, a Last Observation Carried Forward (LOCF) was used. As the pattern of results were largely similar, ITT data for the entire sample was utilized. Post hoc paired t-tests examined differences across the incremental time points. Eta squared examined the magnitude of any significant effects of the intervention across time points (small effect = 0.2, medium effect = 0.5; and large effect = 0.8) [68].

## Results

### Description of the Sample

Three-quarters of the parent sample (*n* = 16, 76.2%) were experiencing elevated parenting stress (≥ 85th percentile) [56] at pre-intervention. In addition, over half of the parents (57.2%, *n* = 12), reported elevated anxiety (≥ 85th percentile on the BSI), [54] and just under half reported elevated depression (42.8%, *n* = 9), and hostility (42.8%, *n* = 9) at pre-intervention.

The mean CSR rating (which ranged from 4 to 8) [64] for the child’s anxiety disorder at pre-intervention was 5.9 (*SD* = 0.8 8). The most common anxiety diagnoses are shown in Table 2. The number of anxiety diagnoses ranged from 1 to 7 and children had an average of 3.25 co-occurring diagnoses (*SD* = 2.09). The most frequent secondary condition, aside from anxiety disorders, was ADHD (10%, *n* = 2), followed by selective mutism (5%, *n* = 1).

**Table 2:** Primary and secondary child diagnoses (n = 21)

Disorder	Primary diagnosis	Secondary diagnosis
	% (N)	% (N)
Separation anxiety	43 (9)	14 (3)
Social anxiety	19 (4)	13 (3)
Specific phobia	19 (4)	24 (5)
GAD^a^	19 (4)	14 (3)

^a^Generalised anxiety disorder

### Baseline Monitoring

Paired samples t-tests comparing the two baseline control assessments for the subsample (*n* = 9) at pre-intervention revealed no significant differences on any parent outcome variables (parenting stress, parent mental health, parent burden, mindful parenting or dispositional mindfulness) over time (all *p* > 0.05). There were also no changes across time on child anxiety severity (*p* > 0.05).

### Adherence

Nine (42%) parents were deemed non-completers as they withdrew prior to completion of five sessions. The remaining 12 (57%) parents were deemed completers, as they completed five or more sessions as well as post-assessment and 3-month follow-up measures. Independent samples t-tests indicated no differences between completers and non-completers on any of the demographic variables. However at pre-intervention, non-completers (*n* = 9) were significantly less depressed (*t*(19) = 2.66, *p* = 0.016), reported less personal distress (PSI-SF) (*t*(18) = 7.84, *p* = 0.019), were less judgmental of themselves as parents-(IM-P) (*t*(18) =  − 3.25, *p* = 0.035), more emotionally aware of themselves (IM-P) (*t*(18) =  − 2.04, *p* = 0.030) and more mindful in their parenting (IM-P), (*t*(18) =  − 11.83, *p* = 0.037) relative to completers.

### Parent Outcomes

The descriptive statistics for parent outcomes at each time-point are shown in Table 3. Repeated measure ANOVAs indicated significant improvements following MPI participation for parenting stress over time (parent–child dysfunctional interaction)*.* Post hoc pairwise t-tests revealed significant reductions for parenting stress from pre-intervention to post-intervention (*t*(20) = 2.86, *p* < 0.005, *d* = 0.62) but not from pre-intervention to 3-month follow-up, or from post-intervention to 3-month follow-up.

**Table 3:** Parent outcomes- intention to treat (n = 21)

Outcome variable	Pre-intervention	Post intervention	3-month follow-up	One Way ANOVA	p	Partial η^2^
	Mean (*SD*)	Mean (*SD*)	Mean (*SD*)	F		
Parent Anxiety	2.91 (1.99)	3.43 (3.50)	3.86 (5.02)	0.388	0.683	0.039
Parent Depression	2.19 (2.60)	1.67 (2.31)	2.14 (2.65)	0.450	0.645	0.045
Parent Hostility	3.67 (2.81)	3.14 (2.10)	3.05 (2.75)	0.532	0.596	0.053
Parent Stress (Total)	95.20 (17.36)	86.90 (20.23)	89.00 (20.39)	3.317	**0.059**	**0.269**
Personal Distress	31.65 (7.71)	29.05 (7.90)	29.09 (6.43)	1.159	0.336	0.114
P/C Dysfunction	25.85 (7.28)	22.62 (7.81)	24.14 (8.023)	4.411	0.027*	**0.317**
Difficult Child	38.66 (8.90)	35.234 (9.62)	35.76 (10.21)	3.182	**0.064**	**0.251**
Parent Burden	42.60 (10.67)	40.39 (11.72)	36.10 (10.28)	2.685	**0.099**	**0.251**
Dispositional Mindfulness	121.70 (19.64)	125.55 (13.41)	130.21 (13.54)	2.246	0.136	0.209
Mindful Parenting	98.35 (11.89)	100.70 (15.27)	104.33 (12.14)	3.769	0.046*	**0.320**

*P/C Dysfunction* parent–child dysfunctional interaction

*p < .05

ANOVA results also confirmed significant improvements following MPI for mindful parenting. Post hoc pairwise t-tests revealed significant effects for mindful parenting from pre-intervention to 3-month follow-up (*t*(17) =  − 2.22, *p* < 0.05, *d* =  − 0.52), and from post-intervention to 3-month follow-up (*t*(17) =  − 2.07, *p* < 0.05, *d* =  − *0.49)*, but not pre- to post-intervention.

ANOVA results indicated that parental mental health (depression anxiety and hostility), overall parenting stress, parenting stress (personal distress and difficult child), parent burden and dispositional mindfulness did not change significantly following the MPI.

Small-medium effects were observed (from pre-post) for mindful parenting, parenting stress (parent–child dysfunctional interaction), parenting stress (difficult child), parent burden and dispositional mindfulness, but were negligible for anxiety, depression, hostility and parenting stress (personal distress). At 3-month follow-up the effect sizes remained small-medium, except for burden which increased to a medium-large effect (*p* < 0.01, *d* = 0.60). ANOVA results showed trends towards improvement in total parent stress, parent stress (difficult child), and parent burden with results approaching significance.

### Child Outcomes

The descriptive statistics for child outcomes are shown in Table 4. The repeated measure ANOVA indicated improvements following parental MPI, with statistically significant decreases in parent-rated total child anxiety symptoms. Post hoc paired t-tests revealed significant reductions in child anxiety symptoms from pre-intervention to post-intervention (*t*(18) = 3.14, *p* < 0.05, *d* = 0.72), and from pre to 3-month follow-up (*t*(20) = 3.85, *p* < 0.01, *d* = 0.84), but not from post to 3-month follow-up.

**Table 4:** Child outcomes- intention to treat (n = 21)

Outcome variable	Pre-intervention Mean (*SD*)	Post intervention N Mean (*SD*)	3 month Follow up N Mean (*SD*)	One Way ANOVA F	p	Partial η^2^
Anxiety Symptoms^a^	61.33 (7.72)	57.67 (9.83)	55.89 (8.69)	7.685	0.004**	**0.475**
Primary anxiety diagnosis severity^b^	5.90 (0.88)	4.70 (1.77)	2.60 (2.76)	8.645	0.009**	**0.684**
Number of comorbid diagnoses^c^	3.62 (2.08)	1.85 (1.95)	1.250 (1.28)	5.030	0.039*	**0.557**

^a^Total score on SCAS = Spence Child Anxiety Scale/PAS = Pre-schooler Anxiety Scale

^b^Clinician Severity Rating (CSR) for Primary Diagnosis from the Anxiety Diagnostic Interview Scale (ADIS)

^c^Total number of child diagnoses from the ADIS

*p < .05; **p < .005

The ANOVA revealed a significant improvement in clinician-rated primary child anxiety diagnosis severity (CSR ratings). Post hoc paired t-tests revealed significant effects for primary child anxiety diagnosis severity from pre-intervention to post-intervention (*t*(12) = 2.70, *p* < 0.01, *d* = 0.75), pre to 3-month follow-up (*t*(11) = 3.57, *p* < 0.05, *d* = . 96) and post to 3-month follow-up (*t*(9) = 2.85, *p* < 0.01, *d* = 0.90).

The ANOVA revealed significant improvements for the number of child co-occurring diagnoses. Post hoc pairwise t-tests revealed significant reductions in the number of child comorbid diagnoses from pre-intervention to post-intervention (*t*(12) = 2.62, *p* < 0.01, *d* = 0.72), from pre-intervention to 3-month follow-up (*t*(11) = 3.94. *p* < 0.01, *d* = 1.1) and from post-intervention to 3-month follow-up (*t*(9) = 2.91, *p* < 0.01, *d* = 0.92).

Effect sizes ranged from medium-large for total child anxiety symptoms, primary child anxiety diagnosis severity, and number of comorbid child diagnoses. At 3-month follow-up these improvements were all maintained and increased to large effect sizes for parent rated anxiety symptoms (*p* < 0.001, *d* = 0.84), clinician rated primary diagnosis severity (*p* < 0.01, *d* = 1.03) and number of comorbid diagnoses (*p* < 0.01, *d* = 1.14).

## Discussion

This novel pilot study is the first we know of to investigate the effectiveness of a MPI for parents of young children with clinical anxiety. The preliminary results support the hypothesis that MPI attendance would significantly reduce parenting stress (parent–child dysfunctional interaction only) at post-intervention and 3-month follow-up, and burden at 3-month follow-up. Contrary to the hypothesis, there was no improvement in parental mental health (anxiety, depression, hostility) at post-intervention or at 3-month follow-up. As predicted, the MPI significantly improved mindful parenting, but not the dispositional mindfulness of parents. The hypothesis that the MPI would be associated with a significant reduction in parent-rated child anxiety symptoms, clinician-rated severity of the child’s primary diagnosis and comorbidity at post-intervention and at 3-month follow-up was also supported.

The finding that the MPI was associated with medium effect size increases in mindful parenting is consistent with those found previously amongst samples of parents of young children with other clinical presentations including autism [45] and ADHD [69, 70]. This MPI appears effective, in terms of increasing mindful parenting for parents of young children with clinical anxiety and may offer strategies that assist them to cope with the caregiving experience.

However, the MPI had only a negligible effect on parents’ dispositional (general) mindfulness. An explanation for this might be that parents increased their skill of noticing of their moment-to-moment experiences in parent–child interactions, but not their general moment-to-moment experiences. It might be difficult for beginners to alter their dispositional mindfulness in a short timeframe. Whereas they may have been motivated (and were directed as part of their home practice) to act mindfully when interacting with their clinically anxious child.

The MPI was associated with significant decreases in (only) one of the parenting stress subscales and a non-significant trend towards decreased burden which increased to a large effect size at 3-month follow-up. Specifically, there was a small non-significant effect for overall parenting stress and burden, and a medium significant effect in the parenting stress domains of (1) parent–child dysfunctional interaction (i.e., decreased parental dissatisfaction with the parent–child relationship and an increased sense that the child is acceptable) and a medium non-significant effect for (2) difficult child (i.e., decreased sense that the child is difficult to parent and unable to regulate their own emotions).

This preliminary research adds to previous evidence supporting MPI’s effectiveness in reducing parental stress and burden in parents of clinical child populations [70]; and extends it into the realm of clinical child anxiety. Mindful parenting practices may offer strategies to assist parents to cope with their own emotions, respond to their own needs, enhancing their caregiving experience. Decreased parental stress, and increased parental acceptance and nonjudgment might translate to higher quality maternal caregiving (i.e., greater warmth, responsiveness), which is predictive of lower subsequent rates of anxiety disorders or remission in children [7, 29, 71]. Indeed, longitudinal studies have revealed that sensitive, maternal caregiving predicts lower rates of anxiety disorders in 4 to 6-year-old children, suggesting that these factors play an important protective role at this developmental stage [29, 30].

Unexpectedly the MPI did not significantly alter parental depression, anxiety or hostility, whereas previous studies have provided preliminary evidence for mindfulness-based interventions significantly improving parental mental health amongst parents of children with ADHD and developmental delays [46–48, 72]. The lack of response in the current study may be explained by several factors. Possibly, this parent population presents with increased vulnerability to anxiety or depression which may be more resistant to change via MPIs, although, without clinical interviews it is difficult to speak to the nature of parent’s mental health status. It might also be that this population requires a more intensive, tailored or individual treatment for anxiety and depression (e.g., CBT). Given that the MPI alone did not sufficiently reduce the parent’s own psychopathology, other evidence-based treatments such as CBT or exposure therapy should continue to be encouraged.

In terms of child outcomes, significant reductions (medium-large effects) were found for parent-rated child anxiety symptoms, clinical-rated primary diagnosis severity and comorbidity, at post-intervention and at 3-month follow-up. These findings are consistent with a previous study [77] which found that children’s internalizing and externalizing problems improved after a MPI. Meppelink et. al. [78] also found that improvements in mindful parenting predicted improvements in children’s symptoms across a range of diagnoses (29% on the autism spectrum, 24% ADHD, 3% anxiety disorder, 1% oppositional defiant disorder and 26% parent–child interaction problem). Our findings are contrary to previous research with a community sample whereby internalising symptoms reduced amongst school age children but did not for children aged 3–5 years [51]; however, should be noted that community samples have less room for improvement than clinical samples.

The current results regarding the MPI reducing child anxiety compliment findings from other parent-based treatment strategies for parents of children with anxiety disorders: Supportive Parenting for Anxious Childhood Emotions (SPACE) [73]. This program identifies family accommodation behaviors and develops strategies to reduce family accommodation as well as manage their child’s responses to the reduced accommodation. Their comparison of SPACE to CBT in a noninferiority trial showed that targeting family accommodation was equally effective as child-based CBT in reducing child anxiety. Additionally, preliminary findings from a qualitative study with anxious parents (N = 10) of anxious children showed that a CBT transdiagnostic parent-based intervention that targeted anxiety-maintaining parenting behaviours and cognitions was considered effective for reducing parental anxiety and coping with bidirectional anxiety maintaining factors for the pair [35]. Taken together, these findings suggest that parent-focused treatment for childhood anxiety disorders may be an effective avenue for future research.

## Limitations and Future Directions

Several limitations of the present study should be considered. Firstly, there was no control condition therefore it was not possible to compare effects of intervention to a randomised waitlist/control. Nevertheless, a baseline group for whom no differences in outcomes were observed across an 8-week period prior to the start of the intervention, functioned as some degree of experimental control.

Secondly, the level of non-completion (43%) was relatively high, although this figure was not dissimilar to other parenting programs which have found attrition to be as high as 51% [74]. One explanation is that parents received sufficient support from several sessions, determined that the intervention was not helpful/irrelevant, or they experienced barriers to attendance (e.g., no childcare, travel time, investment of time), which should be examined in future research to ensure relevance and accessibility to parents. The high attrition may be related to the intervention length and thus briefer, in person, online and self-directed versions of MPI’s deserve further study.

A third limitation was the small sample size that may have resulted in statistical analyses being underpowered to find clinically meaningful effects. In addition, the current study predominantly included mothers, intact families, well- educated parents, and hence the effects remain to be seen for a more diverse sample, amongst whom child rearing commitments and lack of support might pose an even greater barrier to attendance. Hence there is a need for well-designed, fully powered RCT’s with more diverse and inclusive samples to enable generalization of the findings.

Finally future research could supplement self-reported parent outcomes and parent-reported child outcomes with reports from other sources, such as a diagnostic interview with parents and reports from class teachers. Observations of parenting behaviour might examine the connection more reliably between parent-report of mindful parenting and observed parent–child interactions, although there are difficulties observing mindful parenting [75]. Additionally, future studies could also evaluate predictors, mediators and moderators of outcomes for both parent and child outcomes, to determine for whom this intervention is likely to be most effective, as well as elucidate mechanisms of change.

However, this study has some notable unique strengths. This study was the first MPI study of parents of young children with a definitive diagnosis of an anxiety disorder. Second, the sample was characterised by a prevalence of elevated parental stress (76.2%), anxiety (57.2%) and depression (42.8%) which indicate that the MPI is applicable and may be effective for parents of young children with clinical anxiety; however, may need to be adapted to ameliorate parental mental health difficulties. Thirdly, a baseline assessment of outcomes across an 8-week period prior to the start of the intervention was utilized.

## Summary

This pilot study of a mindful parenting approach meets calls to improve implementation and enhance outcomes for highly stressed parents and young children’s mental health needs simultaneously. Anxiety is the most common mental health concern in children and adolescents, and without appropriate intervention increases the risk of poor educational, social and behavioural outcomes and mental health across the lifespan [76]. In sum, this study highlights the preliminary effectiveness of a MPI for parents and for young children with clinical anxiety disorders and describes several avenues for future research and practice.
